# Telesurgery's potential role in improving surgical access in Africa

**DOI:** 10.1016/j.amsu.2022.104511

**Published:** 2022-08-30

**Authors:** Aashna Mehta, Wireko Andrew Awuah, Abdullahi Tunde Aborode, Jyi Cheng Ng, Katherine Candelario, Ines Margarida Pinto Vieira, Halil Ibrahim Bulut, Abdul-Rahman Toufik, Mohammad Mehedi Hasan, Vladyslav Sikora

**Affiliations:** a University of Debrecen, Debrecen, 4032, Hungary; b Sumy State University and Toufik's World Medical Association, Sumy, Ukraine; c Healthy Africans Platform, Research and Development, Ibadan, Nigeria; d Faculty of Medicine and Health Sciences, University of Putra Malaysia, Serdang, Malaysia; e Yale University, Division of Cardiac Surgery, Clinical Outcome Research Group, United States; f University Ovidius of Constanta, Romania; g Istanbul University Cerrahpasa, Cerrahpasa School of Medicine, Istanbul, Turkey; h Department of Biochemistry and Molecular Biology, Mawlana Bhashani Science and Technology University, (MMH), Tangail, Bangladesh

**Keywords:** Telemedicine, Surgery, Health policy

## Abstract

An estimated five billion people worldwide lack access to surgical care, while LMICs including African nations require an additional 143 million life-saving surgical procedures each year.African hospitals are under-resourced and understaffed, causing global attention to be focused on improving surgical access in the continent. The African continent saw its first telesurgery application when the United States Army Special Operations Forces in Somalia used augmented reality to stabilize lifethreatening injuries.Various studies have been conducted since the first telesurgery implementation in 2001 to further optimize its application.In context of a relative shortage of healthcare resources and personnel telesurgery can considerably improve quality and access to surgical services in Africa.telesurgery can provide remote African regions with access to knowledge and tools that were previously unavailable, driving innovative research and professional growth of surgeons in the region.At the same time, telesurgery allows less trained surgeons in remote areas with lower social determinants of health, such as access, to achieve better health outcomes. However, lack of stable internet access, expensive equipment costs combined with low expenditure on healthcare limits expansive utilization of telesurgery in Africa. Regional and international policies aimed at overcoming these obstacles can improve access, optimize surgical care and thereby reduce disease burden associated with surgical conditions in Africa.

## 1 Introduction

An estimated five billion people worldwide lack access to surgical care, while LMICs require an additional 143 million life-saving surgical procedures each year [1]. This disparity can be attributed to several factors. A significant shortage of specialists, which is unique to Africa, severely limits access. In fact, Africa has the fewest specialist surgeons, with only 0.5 surgeons per 100,000 people [2]. Furthermore, 93% of the Sub-Saharan Africa (SSA) population do not have access to surgical care [1]. Most African hospitals are under-resourced and understaffed, causing global attention to be focused on improving surgical access [1,3].

The African continent saw its first telesurgery application when the United States Army Special Operations Forces in Somalia used augmented reality to stabilize life-threatening injuries, provide aid during important limb surgeries, or teach new techniques that allow for faster recovery during the surgery [4]. Telesurgery has the potential to improve surgical access in Africa by eliminating the need for long-distance travel, improving surgical collaborations, reducing surgeon shortages, and lowering infection risk, particularly in tropical regions with frequent infectious disease outbreaks [3,5].

This unique technology connects geographically separated surgeons and patients using advancements in telecommunication and robotic technologies [3]. It also allows supervisors in remote areas to converse real-time with the operating surgeons. This interaction indirectly provided guidance to inexperienced surgeons, improving their confidence and solutions to manage complications that arise during the surgeries. The use of wireless networking, advanced computerized technology, and 3D-high-definition cameras, which provide better visualization and accuracy during surgery, are among the technical advantages of telesurgery [5]. Furthermore, telesurgery procedures improve the outcomes of minimally invasive surgeries by shrinking the incision, reducing scar formation, and hastening the recovery process. It also lowers the risk of common postoperative complications like hemorrhage and infections [3].

Various studies have been conducted since the first telesurgery implementation in 2001 to further optimize its application [3,5]. However, due to global disparities in the burden of surgical diseases and access to surgical care between HICs and LMICs, integration of telesurgery in LMICs has remained difficult. This editorial highlights the potential benefits of telesurgery implementation in Africa, as well as the barriers to telesurgery integration and potential recommendations to improve telesurgical access.

## 2 The current state of the surgical capacity in Africa

African countries have been making efforts to improve their surgical service delivery. AfroSurg Collaborative (AfroSurg), an African-led network established in 2020, serves to identify essential knowledge required to improve surgical policy and service delivery in Africa for all [6]. For instance, the National Department of Health Technical Working Group for surgical services was also established in early 2020, with a long term aim to construct and execute a National Surgical Obstetric and Anaesthesia Plan (NSOAP) for South Africa. The technical working group consists of surgeons, anaesthesiologists, obstetricians, critical care specialists, emergency medicine physicians and other surgical stakeholders from various institutions in South Africa [7]. Both networks hope to improve delivery of an affordable, accessible and comprehensive surgical service.

Despite the limited resources and funding, Africans have published several quality studies, and some even include large cohorts of surgical patients. There are unique surgical pathologies in Africa that could contribute to the knowledge of surgical diseases, and efforts are being made to improve African research outputs. For instance, the South African College of Surgeons has mandated a research report alongside the Masters in Medicine exit examination, before one could qualify as a general surgeon [8].

However, there is still a significant gap in surgery between Africa and other parts of the world. Lack of infrastructure and resources, insufficient surgical workforce, limited surgical care access, and insufficient data place a significant strain on surgical specialties in Africa [2,9]. A prospective observational cohort study involving 25 African LMICs has found that patients receiving surgery in Africa have lower risk profile and complication rates, however their postoperative mortality rate doubled the global average, with infection as the most common postoperative complication [9].

Africa has the fewest surgeons per capita, with an estimated 0.5 surgeons per 100,000 population, compared to HICs, which have an estimated 56.9 surgeons per 100,000 population [10]. The continent's care delivery is hampered by its lack of manufacturing capacity, with the majority of surgical supplies and instruments being imported, limiting their availability to provide safe, timely, and affordable surgical care [9]. Surgical development is currently a low priority in Africa, as evidenced by existing national health plans that barely mention surgery. According to a systematic review, nearly one-fifth of the National Health Strategic Plans in the SSA did not mention surgery or surgical diseases, and nearly one-third of the health policies had no surgical care targets [11].

Other issues of concern include a lack of health education in the community, cultural aspects, and physician burnout in the context of resource and personnel shortages. Because surgical care is a multidisciplinary clinical service, it is critical to address organizational and equipment deficiencies at the primary, secondary, and tertiary levels of healthcare [12]. Finally, low investment and a scarcity of high-quality technology limit the scope of surgical research and training. Nonetheless, it could be argued that high-quality, well-designed study designs can compensate for low technology and produce comparable results.

## 3 Potential benefits of telesurgery implementation in Africa

There is currently little data available on African centers that are implementing telesurgery or conducting related research. Telesurgery is required in all parts of Africa because it eliminates the need to continually increase expenditure to care for some patients [13]. A surgeon, whether in training or not, or a healthcare provider can be guided on how to perform life-saving and defining procedures that have not yet reached those locations using telesurgery. As a result, telesurgery can be especially beneficial in politically conflicted areas such as Sierra Leone, where trauma accounts for 7% of disease burden and an estimated 325,000 deaths. This may be explained by the country's high need but only two specialist surgeons [14,15]. Telemedicine has previously been incorporated in SSA to various clinical services such as psychiatry, endocrinology, pediatrics, and toxicology to name a few. Collective evidence demonstrates it has considerably improved access and quality of care provision as well as case backlog during the pandemic era [16].

Furthermore, mobile phone use in Africa has increased rapidly, rising from 16% in the late 1990s to more than 90% in 2011. Even though internet connectivity is only 15%, the progress is encouraging Africa to consider incorporating information and communications technology (ICT) into surgical service delivery [9]. Telementoring in surgical training is one example of this. A telementoring program aimed at gynaecologic oncologists in the African Republic of Mozambique has improved their knowledge of staging, surgical technique, and preoperative and postoperative patient care [10]. Therefore, similar benefits can be derived from telemedicine integration to surgical services in underserved areas in Africa especially in the context of the current pandemic which exacerbates the prevalent shortage of resources and healthcare personnel [17].

In addition, telesurgery can provide remote African regions with access to knowledge and tools that were previously unavailable. Surgical teams are sometimes too small and require the assistance of specialists who have had more exposure to specific procedures and cases. Telesurgery allows surgeons with less training and in remote areas with lower social determinants of health, such as access, to achieve better health outcomes. Medical personnel caring for burn patients, others who need to quickly relieve increased intracranial pressure, temporarily close a cavity, and those accessing deep vessels to stop bleeding in trauma, among others, can all benefit from advice [15]. A specialist in the field would be able to not only observe everything that a medical provider in a remote area saw and did, but also converse with them in real-time. This interaction may also help to resolve complications that arise during emergency surgeries, especially when inexperienced or non-surgical healthcare professionals are treating emergent cases in underserved African areas [14].

## 4 Addressing potential challenges to telesurgery integration in African healthcare

**Fig. 1:**
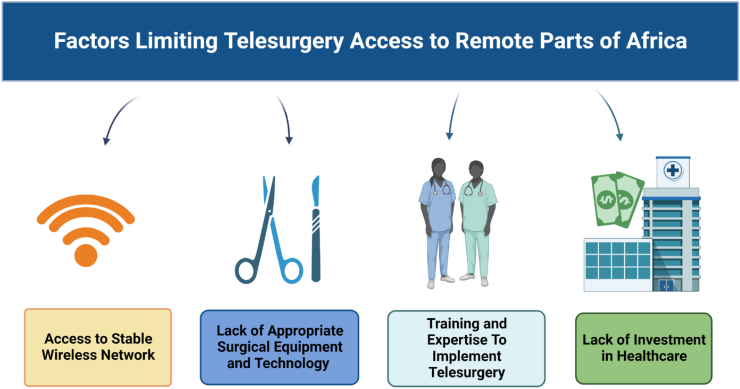
A schematic representation of challenges limiting telesurgery integration in remote parts of Africa. *Created using**biorender.com*.

Telesurgery appears to be a promising solution to Africa's surgical shortage; however, several factors, including a lack of infrastructure, equipment, and socioeconomic issues, limit its implementation(Fig. 1). Purchasing and installing a robotic surgery unit costs millions of dollars while instruction and supporting internet infrastructure cost approximately several hundred thousand dollars per center, which poses a significant economic barrier to access [18].

In addition, over 600 million Africans do not have stable internet access. Lack of stable internet can result in a considerable delay between a surgeon's input instruction and the action implemented by the robotic equipment during the surgery [19]. In telesurgery, even the slightest of delays can severely damage patient tissue and increase the risk of surgical complications. Infact, evidence suggests that a latency of more than 300 ms can significantly deteriorate surgical performance. This limits application of telesurgery over longer ranges especially in transcontinental calls which require effective and reliable reaction time [8,20].

The relatively volatile geopolitical environment can also make ensuring secure and private data processing and meeting legal obligations related to telesurgery difficult [21]. Finally, there is a relative scarcity of data and literature on telesurgery integration in Africa, implying that the region has a low number of telesurgery procedures reported. Furthermore, there are several possible region-specific challenges obscured by a lack of reporting and research on telesurgery implementation that merit further investigation.

## 5 Recommendations

To improve the chances of integrating telesurgery into the African healthcare system, multidisciplinary efforts should be made to bridge the surgical care gap between Africa and the rest of the world. Strengthening National Health Strategic Plans in accordance with the WHO framework and including surgery as one of the main targets is critical to improving access to quality surgical care and, as a result, the development of other surgical advances, such as telesurgery, across African countries. Before incorporating telesurgery into their practices, African countries should devise a step-by-step strategy to improve surgical service delivery. The continent also needs to improve its capacity for manufacturing medical and surgical supplies, which would result in cost savings.

Aside from training surgical personnel, it is also critical to improve the knowledge of the current workforce. It would be advantageous to collaborate with high-income countries through high-quality telementoring or physical programs. Governments should consider incorporating ICT into their healthcare infrastructure. Integrating telesurgery into the system requires a stable internet and electricity supply. Governments should also encourage funding and grants for research into the technicalities of telesurgery implementation. Reducing telesurgery equipment and installation costs while maintaining telesurgery care quality is also important in the continent. Along with the readiness of its infrastructures, telesurgery education and training programs for surgeons could be introduced later, particularly in remote areas with limited personnel. To better guide future healthcare investments, researchers could focus on identifying region-specific barriers to telesurgery as well as potential solutions to overcome them at the global level.

## 6 Conclusion

Overall, regional and international policies aimed at facilitating the incorporation of telesurgery while addressing challenges specific to African countries can be critical in its implementation to advance surgical care. Improved internet access and healthcare investment can lay a solid foundation for the introduction of telesurgery into clinical practice. Furthermore, educational and vocational training in the most recent surgical developments can be critical to the professional growth and finesse of young surgeons. The potential benefits of telesurgery outweigh the current barriers to its widespread use; overcoming these obstacles can improve access and transform surgical care for both patients and healthcare professionals.

## Ethical approval

N/A.

## Sources of funding for your research

N/A.

## Author contribution

AM, WAA, ATA Conceptualized the topic, coordinated reading, writing and editing: AM, WAA, ATA, JCN, KC, IMPV, HIB, ART, MMH contributed to reading, writing, editing the original draft and critical revision: AM, WAA, ATA, JC, MMH contributed to various aspects of reading, data collection, writing the original draft and implementing changes for critical revision under the supervision of WAA, AM, and VS.

## Registration of research studies

Name of the registry: N/A
Unique Identifying number or registration ID: N/A
Hyperlink to your specific registration (must be publicly accessible and will be checked): N/A

## Guarantor

Mohammad Mehedi Hasan.

Department of Biochemistry and Molecular Biology, Faculty of Life Science, Mawlana Bhashani Science and Technology University, Tangail, Bangladesh; mehedi.bmb.mbstu@gmail.com (MMH)

## Consent

N/A.

## Declaration of competing interest

N/A.
